# The first representatives of the millipede family Glomeridellidae (Diplopoda, Glomerida) recorded from China and Indochina

**DOI:** 10.3897/zookeys.954.54694

**Published:** 2020-07-29

**Authors:** Weixin Liu, Sergei Golovatch

**Affiliations:** 1 Department of Entomology, College of Agriculture, South China Agricultural University, 483 Wushanlu, Guangzhou 510642, China South China Agricultural University Guangzhou China; 2 Institute for Problems of Ecology and Evolution, Russian Academy of Sciences, Leninsky pr. 33, Moscow 119071, Russia Russian Academy of Sciences Moscow Russia

**Keywords:** DNA barcode, evolution, *
Glomeridella
*, key, molecular phylogram, new diagnosis, new species, new transfer, *
Tonkinomeris
*, *
Typhloglomeris
*, Vietnam, zoogeography

## Abstract

A new species of glomeridellid millipede is described from Guizhou Province, southern China: *Tonkinomeris huzhengkuni***sp. nov.** This new epigean species differs very clearly in many structural details, being sufficiently distinct morphologically and disjunct geographically from *T. napoensis* Nguyen, Sierwald & Marek, 2019, the type and sole species of *Tonkinomeris* Nguyen, Sierwald & Marek, 2019, which was described recently from northern Vietnam. The genus *Tonkinomeris* is formally relegated from Glomeridae and assigned to the family Glomeridellidae, which has hitherto been considered strictly Euro-Mediterranean in distribution and is thus new to the diplopod faunas of China and Indochina. *Tonkinomeris* is re-diagnosed and shown to have perhaps the basalmost position in the family Glomeridellidae. Its relationships are discussed, both morphological and zoogeographical, within and outside the Glomeridellidae, which can now be considered as relict and basically Oriental in origin. Because of the still highly limited array of DNA-barcoding sequences of the COI mitochondrial gene available in the GenBank, the first molecular phylogenetic analysis of Glomerida attempted here shows our phylogram to be too deficient to consider meaningful.

## Introduction

The chiefly Holarctic millipede order Glomerida (Shelley and Golovatch 2011) is currently known to comprise only three families: Glomeridellidae Cook, 1896, Protoglomeridae Brölemann, 1913, and Glomeridae Leach, 1815 (Enghoff et al. 2015). The family Glomeridellidae presently contains only two accepted genera: *Glomeridella* Brölemann, 1913, with seven or eight species from Spain, France, the eastern Alps, and the northwestern Balkans, and *Typhloglomeris* Verhoeff, 1898 (= *Albanoglomus* Attems, 1926, synonymized by Golovatch (2003)), with ca 15 species, many troglobionts, from the Balkans, Caucasus, and Near East. The family has hitherto been considered strictly Euro-Mediterranean (Enghoff et al. 2015). The ranges of both *Glomeridella* and *Typhloglomeris* overlap only marginally in the northern Dinaric Mountains, Balkans. Makarov et al. (2003) delimited several species groups within *Typhloglomeris* and, based on morphological evidence alone, outlined the main trends of their evolution, both morphological and ecological (= shifts to geo- or cavernicoly).

Continental China, unlike the Nearctic + Southeast Asia + Taiwan which contain several genera of Glomeridae (11) and Protoglomeridae (1) (Enghoff et al. 2015; Nguyen et al. 2019), has heretofore been known to support only numerous (32) species of a single genus, *Hyleoglomeris* Verhoeff, 1910, family Glomeridae (Golovatch and Liu 2020). This genus presently contains 100+ species ranging from the Balkans in the west, through Greece, Anatolia, Caucasus, and Central Asia, to Korea, Japan, and Taiwan in the east and to Indochina, Indonesia (Sumatra, Java, Borneo, and Sulawesi) and the Philippines in the southeast (Golovatch et al. 2006; Enghoff et al. 2015; Nguyen et al. 2019). One species has recently been described from Eocene Baltic amber (Wesener 2019).

All the more interesting is the discovery of a new species of *Tonkinomeris* in southern China. Moreover, this genus appears to actually belong to the family Glomeridellidae, being formally transferred therein from Glomeridae where it was originally placed. This represents the first formal records of glomeridellids not only in China, but also in entire Asia east of Hyrcania (the Republic of Azerbaijan and northeastern Iran near the Caspian Sea coast). The present paper is devoted to a description of our new species and to a discussion of its morphological, molecular and zoogeographical affinities.

## Material and methods

### Morphological analysis

The underlying material was taken from leaf litter in a protected forest patch and preserved in 95% ethanol. The types are deposited in the Zoological Collection of the South China Agricultural University (SCAU), Guangzhou, Guangdong Province, China. A detailed examination of characters and dissections were performed using a Leica S8 APO stereomicroscope. Line drawings were prepared with a Zeiss Axioskop40 microscope with an attached camera lucida. Photographs of specimens were taken with a Keyence VHX-5000 digital microscope and edited using Adobe Photoshop CS6. The terminology used here largely follows that of Golovatch and Turbanov (2018), with only a few modifications.

### DNA extraction and sequencing

Genomic DNA was extracted from legs and thoracic tissue of the paratype with Qiagen DNeasy Blood and Tissue kit following the manufacturer’s extraction protocol. Fragments of the COI gene were amplified using the degenerate primer pair HCO2198-JJ (AWACTTCVGGRTGVCCAAARAATCA) / LCO1490-JJ (CHACWAAYCATAAAGATATYGG) (Astrin and Stüben 2008). The PCR amplification was performed using a T100™ thermal cycler (BIO-RAD) with a final reaction volume of 25 μL. In addition to the new nucleotide sequence in this study, MT522013, 34 Glomerida and nine non-Glomerida sequences (consisting of four Sphaerotheriida, three Polyxenida and two Polydesmida species) as outgroups were downloaded from the GenBank. All analysed species, Genbank accession numbers and voucher numbers/taxonomy ID were listed in Figure 5.

### Phylogenetic analyses

The sequences were aligned using Clustal W and edited in Bioedit (Hall 1999). The final aligned dataset included 44 COI sequences with 656 positions. Bayesian Inference (BI) analysis was implemented through the on-line CIPRES Science Gateway V.3.3 (Miller et al. 2010). The BI analysis was performed by MrBayes 3.2.6 using the Markov chain Monte Carlo technique (MCMC) (Ronquist et al. 2012). The numbers of generations used amounted to 5,000,000 in the parameters for MCMC. The type of a consensus tree was chosen for all compatible groups. Maximum likelihood (ML) analysis was conducted using IQ-TREE web server (Trifinopoulos et al. 2016) with 1,000 bootstrap replications and under the GTR+G+I model (Pende et al. 2014).

## Taxonomy

Considering the new species described below, the following amended diagnosis of *Tonkinomeris* can be proposed.

**Order Glomerida Leach, 1814**

**Family Glomeridellidae Cook, 1896**

### Genus Tonkinomeris

Taxon classificationAnimaliaGlomeridaGlomeridellidae

Nguyen, Sierwald & Marek, 2019

E4740B6B-BDEE-5964-9112-0A9EC6E4B114

#### Type species.

*Tonkinomeris napoensis* Nguyen, Sierwald & Marek, 2019, from northern Vietnam, by original designation (Nguyen et al. 2019).

Other species included: *T. huzhengkuni* sp. nov., southern China.

#### New diagnosis.

A genus of Glomeridellidae with the caudal margins of several ♂ tergites sometimes modified into small lobes drawn posteriad into small lobes; the caudal margin of the ♂ pygidium is clearly emarginate centrally; the anterior telopods are flattened sagittally, somewhat incrassate, with evident mesal outgrowths on either T3 alone or both T2 and T3; posterior telopods with a trichotele (sometimes rudimentary) on T1, each of T2 and T3 with a caudal process and both forming a rather indistinct apical pincer.

### Tonkinomeris huzhengkuni sp. nov.

Taxon classificationAnimaliaGlomeridaGlomeridellidae

16A678F3-F491-5D5E-91AD-7BEC646B7C22

http://zoobank.org/41BF7A61-D9E7-4B81-9685-4D5AB8B7C071

Figures 1 , 2 , 3

#### Type material.

***Holotype***: ♂ (SCAU TY01), China, Guizhou Province, Tongren City, Jiangkou County, Baishuidong Scenic Area, 27.652873N, 108.795223E, 450 m a.s.l., 25.XI.2019, Zhengkun Hu leg. ***Paratype***: 1 ♀ (SCAU TY02), same data as for holotype.

#### Name.

Honours Mr Huzhengkun, the collector and a millipede fan. A noun in genitive case.

#### Diagnosis.

Differs from *T. napoensis* Nguyen, Sierwald & Marek, 2019, the sole other species of the genus (Nguyen et al. 2019), by the larger body size (> 11 mm vs 9.6 mm), the more numerous ommatidia (at least 6+1 vs 5+1), the glabrous, but caudomedially produced posterior margins of ♂ tergites 8–11 (vs unmodified in both sexes), the vivid, peculiar, sexually dimorphic colour pattern (vs even more vivid, but the same in both sexes), and some structural details of the telopods: the much higher central lobe and the much shorter horns of ♂ syncoxite 19 (vs the opposite), the shape and armament of both telopod pairs, the less strongly reduced, 4-segmented ♂ telopodites 17 (vs 2-segmented), etc. (see Discussion below).

#### Description.

**Figure 1.**
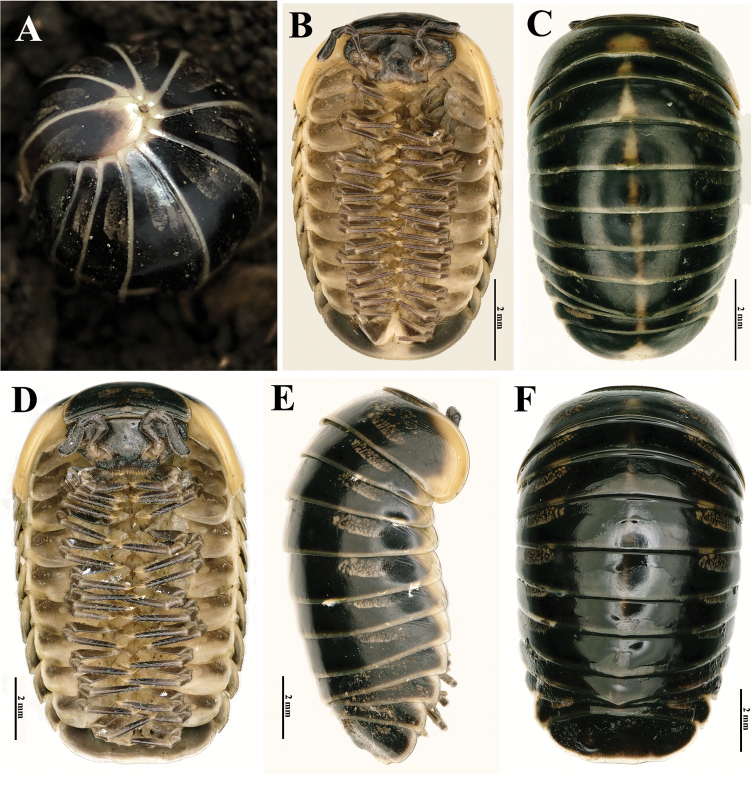
Habitus of *Tonkinomeris huzhengkuni* sp. nov. **A** Live coloration of ♂ holotype **B, C** body of ♀ paratype, ventral and dorsal views, respectively **D–F** body of ♂ holotype, ventral, lateral and dorsal views, respectively.

**Figure 2.**
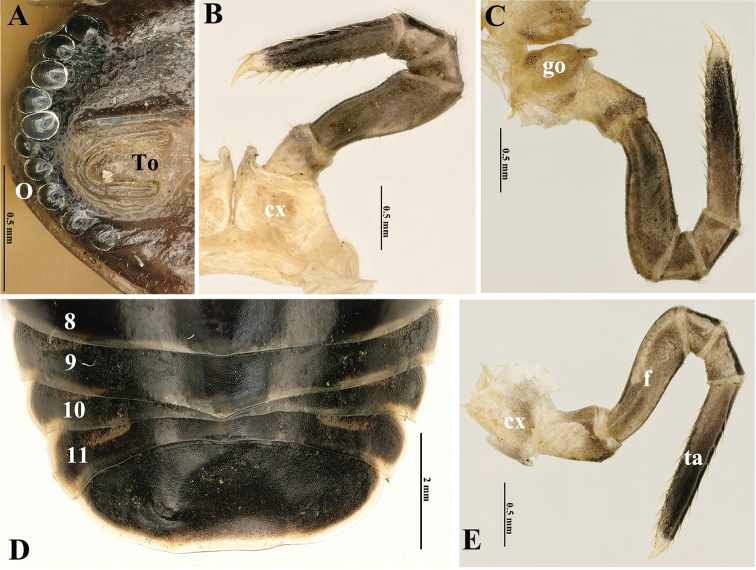
*Tonkinomeris huzhengkuni* sp. nov., ♂ holotype. **A** Right side of head **B** left leg 1 **C** right leg 2 with gonopore **D** posterior part of body, dorsal view **E** left leg 9. Abbreviations: **cx**: coxa, **f**: femur, **go**: gonopore, **O**: ommatidia, **ta**: tarsus, **To**: Tömösváry’s organ, **8–11** refer to tergite numbers.

Body length of holotype ♂ ca 12.5 mm, width of thoracic shield ca 8.0 mm (broadest), height of thoracic shield ca 4.2 mm (highest). Body length of paratype ♀, 11.2 mm, width of thoracic shield ca 7.1 mm (broadest), height of thoracic shield ca 4.0 mm (highest). Coloration (Fig. 1): body rather uniformly blackish, but with slightly yellowish edges. Dorsal pattern marbled yellow-brownish: collum with a small, rounded, central spot anteriorly and a paramedian pair of larger, transversely ovoid, lateral spots (Fig. 1D); thoracic shield and tergites 3–11 each with a similar, but much larger pair of lateral spots and a yellowish, but slightly purplish, median, narrow, axial stripe. Anal shield (= pygidium) of ♂ without stripe (Figs 1F, 2D), but ♀ with a distinct, yellowish, median, triangular spot near caudal margin (Fig. 1C). Head largely dark brown, only labrum and Tömösváry’s organ lighter, grey-yellowish. Antennomeres 3–6 brownish, slightly purplish, remaining antennomeres light yellow-brown. Venter and podomeres 1 and 2 entirely light grey-yellowish, remaining podomeres dark brown, slightly purplish (Fig. 1B, D).

**Figure 3.**
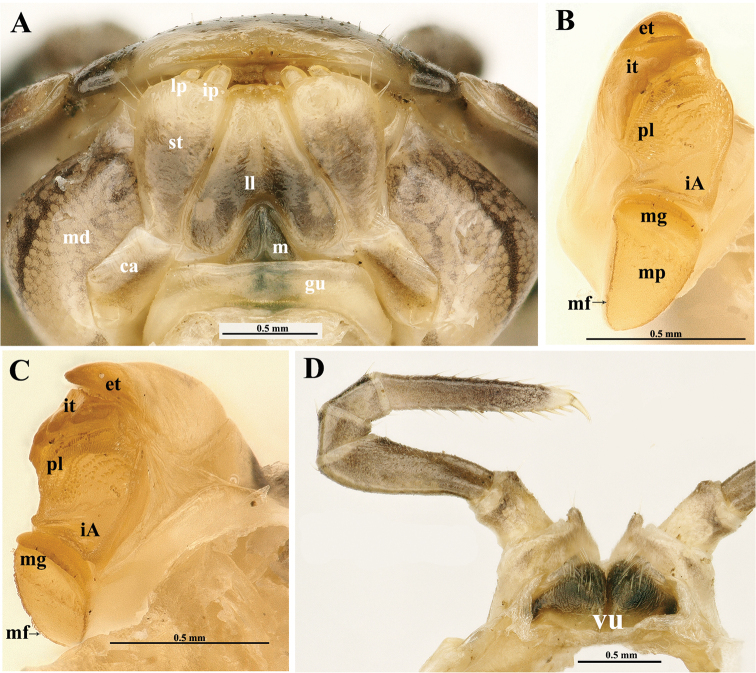
*Tonkinomeris huzhengkuni* sp. nov., ♀ paratype. **A** Gnathochilarium, ventral view, *in situ***B, C** left mandible, mesal and subfrontal views, respectively **D** coxae 2 with vulvae. Abbreviations: **ca**: cardines of gnathochilarium, **et**: external tooth, **gu**: gula, **iA**: intermediate area, **ip**: inner palpus, **it**: inner tooth, **ll**: lamellae linguales, **lp**: lateral palpus, **m**: mentum, **md**: basal joint of mandible, **mf**: membranous fringe, **mg**: molar groove, **mp**: molar plate, **pl**: pectinate lamellae, **st**: stipites, **vu**: vulvae.

***Head***: mandibles (Fig. 3B, C) equal in both sexes, each with a large external tooth and a smaller internal tooth, the latter with four cusps. Molar plate with a long membranous fringe and a groove. At least seven rows of pectinate lamellae and a scaly intermediate area. Gnathochilarium (Fig. 3A) equal in both sexes, unmodified, typical of Glomerida. Left and right eyes asymmetrical, with 9+1/10+1 (♂) (Fig. 2A) or 8+1/6+1 (♀) (Fig. 1B) ommatidia. Tömösváry’s organ transversely oval, ca 1.6 times wider than long (Fig. 2A). Lengths of antennomeres: 6>>3>4=5>1=2>7 (Fig. 1B, D). Antennomere 6 ca 2.6 times as long as high. Antennomere 8 with four small, apical, sensory cones.

***Collum*** with two complete transverse striae (Fig. 1D). Thoracic shield with a narrow hyposchism extending past caudal tergal margin; about 12 or 13 superficial transverse striae laterally and dorsolaterally, but five or six confusedly arranged and incomplete. One or two starting below, one in front of schism, all others above schism, with three crossing the dorsum; mid-dorsal region with five additional, incomplete, broken, confused, mostly short striolae behind last regular stria (Fig. 1E). Tergites: surface smooth and shining, only paratergites with three or four short, incomplete, and superficial striae (Fig. 1E). Tergite 9 in ♂ drawn posteriad into a small, triangular, glabrous (non-dentate), median lobe (Fig. 2D), this being very week also in tergites 8, 10, and 11. Pygidium in both sexes uneven medially at caudal margin; in ♂ clearly impressed and concave centro-dorsally and with two very small, paramedian, flattened and rounded knobs (Figs 1D–F, 2D), in ♀ only slightly flattened dorsocaudally (Fig. 1C).

***Legs*** long and slender. All podomeres densely setose, setae mostly being short. Coxae 1–16 each with a short, well-rounded, spinigerous, apico-mesal projection, this being especially evident in coxae 1 and 2 (Fig. 2B, C). Coxae 4–21 each with a similar apico-lateral process. Tarsi 1–16 each with two irregular transverse rows of 7–8+7–8 dorsal spines and 9–12+9–12 ventral spines (Fig. 2B, C, E). Femur 9, 2.4 times, tarsus 6.5 times longer than wide (Fig. 2E).

**Figure 4.**
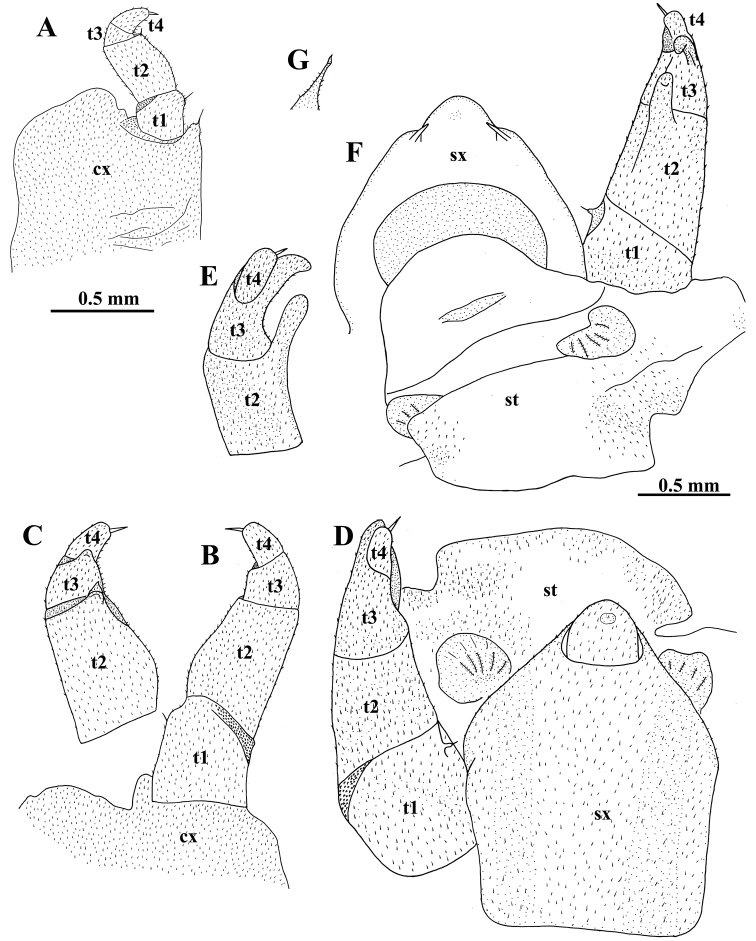
*Tonkinomeris huzhengkuni* sp. nov., ♂ holotype. **A** Left leg17, oral view **B** right anterior telopod, oral view **C** right anterior telopod, caudal view **D–F** left posterior telopod, oral, mesal and caudal views, respectively **G** tip of syncoxital horn, caudal view. Abbreviations: **cx**: coxa, **st**: sternite, **sx**: syncoxite, **t1–t4**: telopoditomeres 1–4. Scale bars: 0.5 mm (**A–C**, left); 0.5 mm (**D–G**, right).

***Male sexual characters***: gonopore small, oval, with a few short setae around (Fig. 2C). Legs 17 (Fig. 4A) strongly reduced, very densely micropilose throughout. Coxae membranous, contiguous, but clearly separated medially. Each coxa with a very large, rather regularly rounded, outer lobe and a small, rounded, distomedial, setigerous finger. Telopodite 4-segmented, telopoditomere 2 largest, subrectangular, about twice as long as telopoditomere 1 or telopoditomeres 3 and 4 combined. ***Anterior telopods*** (Fig. 4B, C) also very densely micropilose throughout. Syncoxite (= ?coxosternum) membranous, on either side with a small rounded lobule at base of telopoditomere 1. Telopodite 4-segmented, with a spine apically. Telopoditomere 1 subrectangular, 1.2 times longer than wide. Telopoditomere 2 largest, a little swollen ventro-parabasally, its apico-mesal tooth on caudal face bulged at base, sharp apically and extending to basal 1/4 telopoditomere 3. The latter subtrapezoid, its apico-mesal tooth on caudal face small, rounded, projecting above base of a subcylindrical telopoditomere 4. ***Posterior telopods*** (Fig. 4D–G) particularly strongly incrassate, likewise very densely micropilose throughout. A large, high, thick, and roundish syncoxite placed on a large membranous sternite, with a high, roundish, median lobe closely flanked by two inconspicuous, short, spiniform, obliquely truncate, setose horns (Fig. 3G), each latter being much shorter than syncoxital lobe. Telopodite 4-segmented, with a spine apically. Telopoditomere 1 squarish, with a very small distomesal, setigerous cone (a strongly reduced trichostele). Telopoditomere 2 with a prominent, finger-shaped, distomesal process on caudal face, produced apically to ca 1/3 telopoditomere 3. The latter elongate, gently tapering distad and curved apically basad towards process on telopoditomere 2, with another, much smaller, caudad curved, caudolateral process. Telopoditomere 4 smallest, subcylindrical, erect, clearly shifted anteriad, subtended by and reaching the distal end of telopoditomere 3.

***Vulva*** (Fig. 3D) densely setose, large, covering 1/2 coxa 2.

## Comparative morphology and systematics

Originally, *Tonkinomeris* was described in the family Glomeridae Leach, 1815, tentatively assigned to the subfamily Haploglomerinae Mauriès, 1971, and compared to the genus *Peplomeris* Silvestri, 1917, with two species from northern Vietnam (Golovatch 1983; Nguyen et al. 2019). Both *Tonkinomeris napoensis* and *T. huzhengkuni* sp. nov. are very similar and are also sufficiently close geographically. They can easily be distinguished by a good number of morphological characters: both show vivid colour patterns (apparently, because both are epigean and fairly large), the caudal margin of the ♂ pygidium is clearly emarginate centrally (yet with no evident paramedian tubercles), T3 is somewhat incrassate and sagittally flattened, the anterior telopod (♂ leg 18) is supplied with a blunt apico-mesal tooth, there is an elongate, subcylindrical, and suberect posterior telopod (♂ leg 19) which features T1 with a mesal trichostele, each of T2 and T3 have a distinct distocaudal process, etc. Furthermore, the telopodites of the posterior telopods are not only 4-segmented and supplied mesally with a trichostele on T1, but they also show a small caudal process on T3 in addition to a stronger caudal process on T2; thus, T3 is well developed, fully functional, and its apical part forms a kind of underdeveloped pincer together with T2. All this allows us to relegate *Tonkinomeris* from Glomeridae to the family Glomeridellidae. Moreover, as the apical pincer on the posterior telopod seems to be a little better developed in *T. huzhengkuni* sp. nov. than in *T. napoensis*, this pincer in the remaining Glomeridellidae may be considered as being clearly apomorphous. This contradicts the views of Oeyen and Wesener (2015) to regard the Glomeridellidae as the basal family of the order Glomerida, better agreeing instead with their later cladistic analysis (Oeyen and Wesener 2018).

In addition, like most species of *Typhloglomeris*, the caudal margins of a few ♂ tergites in front of the pygidium in *Tonkinomeris huzhengkuni* sp. nov. are modified, each drawn medially posteriad into a small, albeit glabrous, lobe (thus, clearly apomorphous), vs remaining simple and unmodified (plesiomorphous) in *T. napoensis*. In contrast, the particularly strongly reduced, 2- or 3-segmented ♂ telopodites 17 in *T. napoensis* definitely represent an apomorphous condition compared to the usual, 4-segmented ♂ telopodites 17 observed in *T. huzhengkuni* sp. nov. and most other Glomerida. The presence of a sharp caudomesal tooth also on T2 of the anterior telopod, vs its absence from *T. napoensis*, is difficult to polarize in terms or apo- or plesiomorphy. However, the particularly strongly developed central syncoxital lobe and the especially small syncoxital horns, as well as the rudimentary trichostele on T1 of the posterior telopods, all observed in *T. huzhengkuni* sp. nov. as opposed to their more usual states in *T. napoensis*, seem to be apomorphous. Therefore, each of the species combines both apo- and plesiomorphies in a number of traits. Most of the characters seem to be more advanced (apomorphous) in *T. huzhengkuni* sp. nov. compared to *T. napoensis*, but a few others vice versa (e.g., the more strongly reduced ♂ legs 17). What appears evident in any case is, that overall *Tonkinomeris* seems to represent the most primitive, perhaps even the basalmost genus of Glomeridellidae. This is primarily because both *T. napoensis* and *T. huzhengkuni* sp. nov. still show very modest modifications of the ♂ tergites and pygidium, while their posterior telopods feature a trichostele on T1 and yet underdeveloped apical pincers formed by T2 and T3.

Glomeridae, in contrast to Glomeridellidae, are distinct in the posterior telopods (♂ legs 19) typically being stouter, clearly curved mesad, by themselves forming a strong pincer, some telopoditomeres before last one showing a mesal trichostele or its vestige, while each telopodite is devoid of clear-cut apical pincers. The Protoglomeridae seems to be a polyphyletic group (Oeyen and Wesener 2015), only superficially being similar to Glomeridae; sometimes their tergite 11 is fused to the pygidium (still retaining a suture), while the posterior telopods are even stouter, devoid of trichosteles, both T2 and T4 form a distinct pincer by themselves (T3 being strongly developed), while each telopodite is with an additional apical pincer due to T2 and T4 (e.g., Mauriès 1971). Among the Glomeridellidae, however, the posterior telopods are usually contrasting elongate, slender, suberect, each telopodite forming a more or less distinct pincer due to modified T2 and T4 or T2 and T3 (Attems 1926; Mauriès 1971; Enghoff et al. 2015). Within *Typhloglomeris*, the genus deemed both morphologically and geographically closest to *Tonkinomeris*, the pincers on the posterior telopods in most species are formed by T2 and T3=4, when the real T3 is completely suppressed, or by T2 and T4, when T3 is strongly reduced to a short, rudimentary, non-functional, but still visible, albeit sometimes incomplete, ring. In contrast, the pincers in *Tonkinomeris* tend to be somewhat underdeveloped and peculiar in showing a small caudal process on T3 in addition to a stronger caudal process on T2, with T3 being fully developed and functional. This definitely represents a plesiomorphy, perhaps even the basalmost state whence a gradual reduction of both T3 and a trichostele on T1 is traced within some more advanced Glomeridellidae like *Typhloglomeris* and *Glomeridella* (cf. Attems 1926; Mauriès 1971).

## Phylogeny

The phylogeny of Glomerida as recently recovered by Oeyen and Wesener (2018), based on morphological evidence alone, shows that both *Glomeridella* and *Typhloglomeris* cluster together with some Protoglomeridae and thus form no clear-cut family Glomeridellidae. Moreover, the joint Protoglomeridae + Glomeridellidae clade is not too basal on the tree and, thus, better agrees with our views that the Glomeridellidae is best considered as one of the relatively advanced groups of Glomerida.

**Figure 5.**
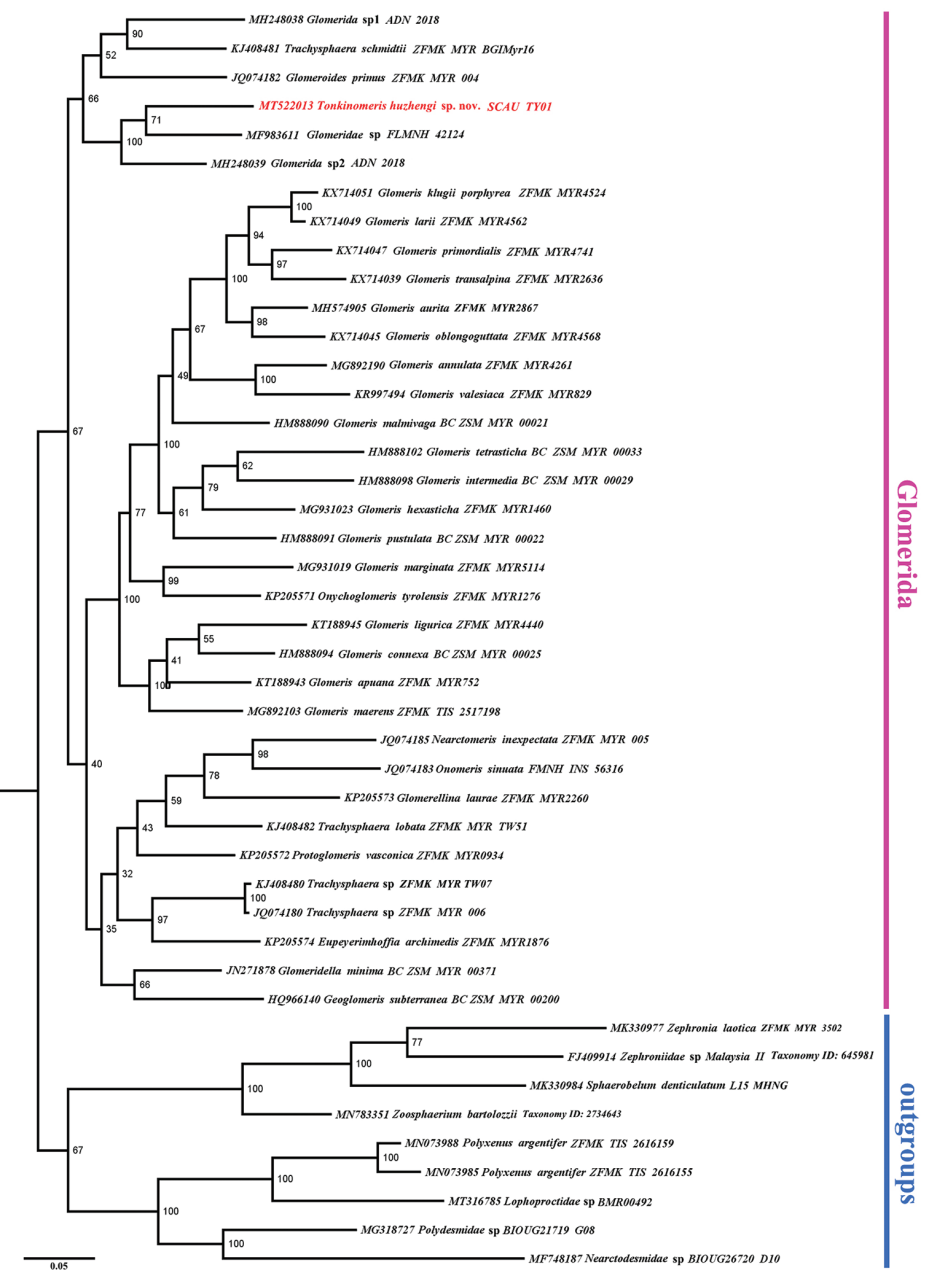
A consensus tree produced from BI analysis. Numbers on branches are estimates of the Bayesian posterior probability of a clade, expressed as percentage.

The molecular sequences available in the GenBank and used in our phylogenetic analysis, which is apparently the first to be attempted for the entire order Glomerida, have allowed for two phylograms to be obtained. Since both BI and ML trees are similar and neither is congruent with the morphology-based phylogeny recovered by Oeyen and Wesener (2018), we present here only the BI tree (Fig. 5). Because the only genetic data available for Glomeridellidae in the GenBank are for a species of *Glomeridella*, and there is nothing yet for any *Typhloglomeris* sp., *Tonkinomeris* appears to cluster together with or close to two closer unidentified members of Glomeridae, one of which seems to be a *Hyleoglomeris* sp. (Wesener pers. comm.). Thus, there is no hint of a Glomeridellidae cluster. Instead, the whole tree (Fig. 5) is a rather random mixture of mostly genera and species of Glomeridae. This seems to indicate that any molecular analysis is bound to be too deficient and premature at this stage. It would seem especially interesting to compare *Tonkinomeris* to some other representatives of Glomeridellidae, especially the morphologically and geographically closest *Typhloglomeris* spp.

## Key to genera of Glomeridellidae

The following key to the accepted genera of Glomeridellidae can be offered:

**Table:** 

1	Penultimate (11^th^) tergite just in front of pygidium strongly reduced, visible only laterally as thin ribbons. Caudal margins both of tergites and pygidium regularly rounded caudally, unmodified. Tergites densely and finely pubescent. Anterior telopods strongly elongate, subcylindrical, T2 and T4 forming a pincer, T3 being small. Syncoxital lobe of 3-segmented posterior telopod telopodites very simple and low, a trichostele on T1 absent, both T2 and T3=4 forming a pincer (apparently, true T3 being totally reduced). Western Europe east to northwestern Balkans	*** Glomeridella ***
–	No tergites reduced. Caudal margin of tergites and pygidium either unmodified and regularly rounded or (in ♂ only) modified. Tergites bare. Anterior telopods relatively stout, mostly flattened sagittally, each forming no apical pincer. Syncoxital lobe of posterior telopods higher and variously shaped, telopodites 3- (more rarely) or 4-segmented, usually elongate, slender, suberect and each forming a more or less distinct apical pincer	**2**
2	Caudal margin of some tergites and pygidium usually modified, several tergites before ♂ pygidium largely crenulate, ♂ pygidium with a paramedian pair of distinct knobs at a centrally emarginate or nearly straight caudal edge. Anterior telopods often flattened sagittally, sometimes also inflated, but usually devoid of mesal outgrowths. Posterior telopods devoid of trichoteles and each forming a distinct apical pincer (either T2 and T4, when T3 rudimentary, or T2 and T3=4, when true T3 fully suppressed). Eastern Mediterranean	*** Typhloglomeris ***
–	Caudal margin of some tergites and/or pygidium modified, several tergites before ♂ pygidium glabrous, not crenulate, but sometimes drawn caudad into small central lobes, while ♂ pygidium with a centrally emarginate caudal margin and only sometimes with a paramedian pair of indistinct knobs at rear edge. Anterior telopods flattened sagittally, with evident mesal outgrowths. Posterior telopods with both a trichostele retained on T1 and an indistinct apical pincer (T2 and T3). Southern China and northern Vietnam	*** Tonkinomeris ***

## Zoogeography

Finding a glomeridellid genus in southern China and northern Vietnam is indeed remarkable, as the geographically closest record belongs to *Typhloglomeris martensi* (Golovatch, 1981), from Hyrcania, southwesternmost Caspian Sea coast within both the Republic of Azerbaijan and northwestern Iran (Golovatch 1981). As the huge gap between Hyrcania and Guizhou Province definitely reflects traces of former extinctions and dispersal events, this allows for the entire family Glomeridellidae to be considered both relict and of Oriental stock. Because on balance *Tonkinomeris* seems to be the most primitive among the glomeridellid genera, this also allows us to suggest some ancient, generally northwestward dispersal events from the Oriental realm to the Mediterranean area via southern China. Interestingly, in certain respects the relatively more advanced *T. huzhengkuni* sp. nov. looks like the remain of a stepping-stone in Guizhou Province, China; this is also quite far west of the overall more primitive *T. napoensis* from Vietnam, near the family’s presumed Oriental roots.

The above picture not only so considerably extends the known distribution area of Glomeridellidae to the east, but it also demonstrates the extent to which the millipede fauna of China is still understudied, as well as the possible roles that the Sino-Himalayan (= southern Chinese) and/or Oriental faunogenetic centres could have played in the origins of the Euro-Mediterranean diplopod fauna (Golovatch and Martens 2018; Golovatch and Liu 2020). Such a distribution pattern strongly resembles that of *Hyleoglomeris*, one of the largest, diverse, and widespread genera of Glomeridae and Glomerida (see above).

More information is necessary, especially phylogenetic reconstructions, in order to assess the remarkable disjunction of the Glomeridellidae and both its biological and spatial evolution. Further conclusions must be deferred until more evidence, both morphological and molecular, becomes available. New Glomerida are still being actively found and described from various places in Asia!

## Supplementary Material

XML Treatment for Tonkinomeris

XML Treatment for Tonkinomeris huzhengkuni
